# Zeolitic-imidazolate frameworks derived Pt-free counter electrodes for high-performance quantum dot-sensitized solar cells

**DOI:** 10.1098/rsos.180335

**Published:** 2018-05-30

**Authors:** Wenjiao Xu, Yuxiu Sun, Bin Ding, Jingbo Zhang

**Affiliations:** Key Laboratory of Inorganic-Organic Hybrid Functional Material Chemistry, Ministry of Education, Tianjin Key Laboratory of Structure and Performance for Functional Molecules, College of Chemistry, Tianjin Normal University, Tianjin 300387, People's Republic of China

**Keywords:** ZIF-67, sulfuration, carbonization, counter electrode, QDSSCs

## Abstract

Zeolitic-imidazole frameworks (ZIFs), as novel porous materials, are attracting much attention in several fields due to their special advantages such as large specific surface area, versatile porosity and well-connected networks. Here, we develop a porous ZIF-derived catalytic thin film, which was coated on the conducting glass as a counter electrode (CE) to substitute costly platinum for quantum dot-sensitized solar cells (QDSSCs). A ZIF layer is first prepared by coating ZIF-67 powders on the conducting glass, followed by the careful calcination treatments in sulfur vapour (sulfuration) or nitrogen gas (carbonization). The structure and morphologies of the derived porous film are characterized by the measurements of XRD, SEM and BET, and the electrochemical properties in the polysulfide solution are evaluated by the measurements of Tafel curves and electrochemical impedance spectroscopies. The derived porous film is used as a CE to fabricate QDSSC with CdSe quantum dot-sensitized TiO_2_ nanocrystalline thin film and the polysulfide solution. Compared with the photovoltaic performance of CdSe QDSSCs based on the CE prepared by the different sulfuration conditions, QDSSC based on the CE derived by the sulfuration for 30 min shows an excellent light-to-electric conversion efficiency of 3.77%, it is even higher than that of QDSSC based on Pt CE (2.98%). This work will open a new avenue to design a facile, low-cost and renewable CE for QDSSC.

## 1. Introduction

Quantum dot-sensitized solar cell (QDSSC), as a most potential application in the field of solar cell in future [1], consists of quantum dot-sensitized nanoporous thin film photoelectrode, electrolyte and counter electrode (CE). As a critical component of QDSSCs, the CE serves to collect electrons from the external circuit and transfer them to the electrolyte to reduce the oxidized electrolyte. Therefore, CE plays an important role in deciding the light-to-electric conversion efficiency of QDSSCs. In the case of polysulfide system, the usually used electrolyte for QDSSCs, the CE catalyses the reduction of ${{\text{S}}_{n}}^{2-}$ to S^2−^ at the CE/electrolyte interface. Here, high catalytic activity and excellent conductivity should be taken into account in searching for a good CE material. Precious metal platinum has been applied over several years; however, it is costly and unrenewable and its catalytic activity towards the polysulfide is poor. Therefore, Pt-free CE catalytic materials for QDSSCs [2,3], such as transition metal sulfide [4], carbon and nitrogen materials (graphite, active carbon, carbon black, single-wall carbon nanotubes, nitrogen-doped porous carbon nanorods, Nb–Ti–N and Ni_2_N) [5–12], PEDOT, polyaniline and PProDOT-Et_2_ [13–15], have been reported. At present, the photoelectric conversion efficiency of QDSSCs is unsatisfied. Although QDSSC has the very significant application prospect due to QDs with the ability to generate multiple charge carriers from a single photon, there is a larger distance towards its practical application [16,17]. Therefore, it is still profound to search the novel low-cost CE with high catalytic activity and controllable microstructure.

As a kind of materials to be most researched, metal-organic frameworks (MOFs) are a type of organic–inorganic hybridized materials, which are formed by organic ligands, metal ions and a cluster of atoms. MOFs have attracted great attention recently in the inorganic and organic field owing to their large specific surface area, high porosity, adjustable pore size, channel rules, versatile porosity and well-connected networks. There are some important applications in the hydrogen storage, gas adsorption and separation, sensors, drug sustained release and catalytic reaction [18–20]. Besides, MOFs are applied in the field of photoelectrochemistry recently. For example, MOFs are used as a conducting layer in the working electrode of dye-sensitized solar cell [21]. MOFs are applied in the late-model perovskite solar cell to upgrade the efficiency and stability [22–27]. A zeolitic-imidazole framework (ZIF) is a well-known subfamily of MOFs. Among them, ZIF-67 has been most studied due to the uncomplicated synthesis, long-term stability and the presenting of transition element cobalt in the framework centre, which has innate conductivity and magnetism. Its carbide and nitride have been also used for the electrode [28]. With the rhombic dodecahedron of zeolite framework and the central element of cobalt, ZIF-67 has a large specific surface area and excellent conductivity. And the introduction of oxygen, sulfur and selenium can change the performance of MOFs [29]. Recently, ZIFs are extensively investigated in supercapacitor, gas storage, membrane separation and catalysis due to their unique features such as permanent porosity and tuneable functionalities [30–33].

Here, we developed an efficient and stable ZIF-67-derived CE for QDSSCs by a simple and effective interface sulfuration and carbonization method. The porous carbon material can be obtained by controlling the carbonization processes of ZIF-67. The hybrid porous carbon with CoS can be prepared by the sulfuration processes. Recently, nanostructured metal sulfides have been used as CEs, such as CoS, CuS and NiS [34]. However, with ZIFs as the precursor, the hybrid material of CoS containing carbon can be formed. The hybrid CE possesses the functions of both CoS and C. The ZIF-derived CEs will lead to better catalytic performance and thus higher light-to-electric conversion efficiency. In this work, the prepared hybrid materials were characterized in detail. The performance of the ZIF-67-derived porous material as CE of QDSSCs was measured to evaluate the catalytic activity of the derived CE.

## 2. Experimental

### 2.1. Materials

Cobalt chloride (CoCl_2_), 2-methylimidazole (MIm), sulfur powder (S), polyvinyl pyrrolidone (PVP), cadmium sulfate (CdSO_4_), nitrilotriacetic acid (NTA), sodium hydroxide (NaOH), selenium powder (Se), anhydrous sodium sulfite (Na_2_SO_3_), titanium chloride (TiCl_4_), sodium sulfide (Na_2_S), zinc nitrate (Zn(NO_3_)_2_), chloroplatinic acid (H_2_PtCl_6_^·^H_2_O), methanol, acetone, ethanol, isopropanol, *n*-butanol and TiO_2_ nanoparticle powders with the average size of 25 nm (Degussa P25) were purchased from Alfa Aesar, Inc., China. The fluorine-doped tin oxide (FTO) conducting glass (10 Ω sq^−1^, Nippon Sheet Glass Co., Ltd, Japan) was ultrasonically washed in deionized water, acetone and ethanol, and finally immersed in isopropanol.

### 2.2. Preparation of the ZIF-67-derived CEs and Pt CEs

The typical synthesis method of ZIF-67 was as follows: 0.935 g of CoCl_2_ was dissolved in 100 ml of methanol. At the same time, 1.376 g of MIm with a small amount of PVP was also dissolved in 100 ml of methanol. And then, two methanol solutions were mixed by stirring and the stirring was continued for 10 min. The mixture was transferred into a Teflon reaction bottle, which was heated at 100°C for 12 h. After centrifugation, the as-prepared ZIF-67 (bright purple powder) was washed with methanol, and then dried in vacuum at 80°C. The addition of PVP during synthesis of ZIF-67 is to control the particle size of ZIF-67. The small ZIF-67 particles will benefit the formation of homogeneous ZIF-67 thin film.

For the electrode preparation, the appropriate amount of ZIF-67 powder was dispersed in *n*-butanol, it was further ground to form a slurry, which was printed onto the FTO conducting glass by the doctor-blade method. The prepared thin film was carbonized at 450°C in N_2_ gas for 15 min (named as C15), 30 min (named as C30) or calcinated under sulfur powder steam for 15 min (S15), 30 min (S30), 60 min (S60), 120 min (S120) and 180 min (S180). The prepared thin films were also calcinated by combining two calcinating methods. It was first carbonized for 15 min and then sulfurated for another 15 min (C15 + S15) or for another 30 min (C15 + S30), as well as sulfurated for 30 min after carbonized for 30 min (C30 + S30). Pt CEs were prepared by dropping 10 µl of 5 mM chloroplatinic acid solution in isopropanol on FTO substrates followed by the thermal decomposition of the H_2_PtCl_6_^·^H_2_O at 390°C for 30 min.

### 2.3. Preparation of CdSe QD-sensitized TiO_2_ nanocrystalline thin films

Nanocrystalline TiO_2_ thin films were prepared by coating P25 TiO_2_ colloids on the FTO conducting glass with the doctor-blade method. Then, the thin films were sintered at 450°C for 30 min, followed by modifying in the 0.5 mM TiCl_4_ solution. CdSe QDs were synthesized by the method of chemical bath deposition to sensitize the TiO_2_ thin film as the working electrode according to the reported method [35,36]. The SILAR approach was employed to deposit ZnS on the surface of CdSe QD-sensitized thin film. ZnS was deposited by placing the electrode in the 0.5 M Zn(NO_3_)_2_ solution for 1 min, then in 0.5 M Na_2_S for another 3 min and repeating it five times. Finally, the electrode was washed with deionized water and dried under an ambient atmosphere.

The QDSSCs were fabricated with the CdSe QD-sensitized TiO_2_ thin film working electrode and a series of MOF-derived CEs or Pt CE for comparison. The electrolyte was 0.5 M S, 2.0 M Na_2_S and 0.2 M KCl dissolved in the mixing solvent of water and ethanol with the volume ratio of 7 : 3.

### 2.4. Characterization of the derived CEs

The crystalline structure of carbonized and sulfurated ZIF-67 was characterized by an X-ray powder diffractometer (XRD, Bruker D8 ADVANCE, Cu Kα). Surface morphologies of the thin films were observed on a scanning electron microscope (SEM, FEI Nova Nano SEM 230, 15 kV). Electrochemical impedance spectroscopy (EIS) was measured in the darkness with a frequency response tracer (Solartron 1255B) and a potentiostat (Solartron SI 1287). The frequency range of EIS is 10^5^–10^−1^ Hz. Both Tafel curves and cyclic voltammetry (CV) curves of CEs were measured on a potentiostat (Hokuto Denko HSV-100). A saturated calomel electrode as a reference and a Pt wire as a counter are placed in the cell. Tafel curves were measured at a constant scan rate of 10 mV s^−1^ and a sensitivity of 100 mV, and CV curves were measured at a scan rate of 5 mV s^−1^ and a sampling interval of 100 ms. Photocurrent–voltage (*J–V*) curves of the assembled QDSSCs were measured on a potentiostat (Hokuto Denko HSV-100). The active area of the cell was 0.2 cm^2^, which was illuminated with a solar simulator (Newport 94023A) under the simulated AM 1.5 sunlight (1 sun, 100 mW cm^−2^).

## 3. Results and discussion

### 3.1. Characterization of ZIF-67-derived thin films

MOFs, as crystalline porous materials with the periodic network, are formed by transition metal cations and organic ligands through self-assembly. Metal ions generally act as the connecting point to support the three-dimensional pore structure, and the organic ligands extend the structure space. If MOFs are carefully calcinated under inert gas, their porous structure can survive after organic ligands except carbon are burned off. The left carbon porous materials have been reported to have good catalytic activity as the CE of dye-sensitized solar cell [37]. If MOFs are calcinated under sulfur stream, metal ions in the MOF structure can react with sulfur to form metal sulfide. The transition metal sulfide with specific structure has been certificated to be a good CE for QDSSCs [4]. Therefore, we try to control the calcinating condition of MOFs under both N_2_ gas and sulfur stream to form porous structure of carbon and metal sulfide, which will be used as the CE for QDSSCs.

**Figure 1.**
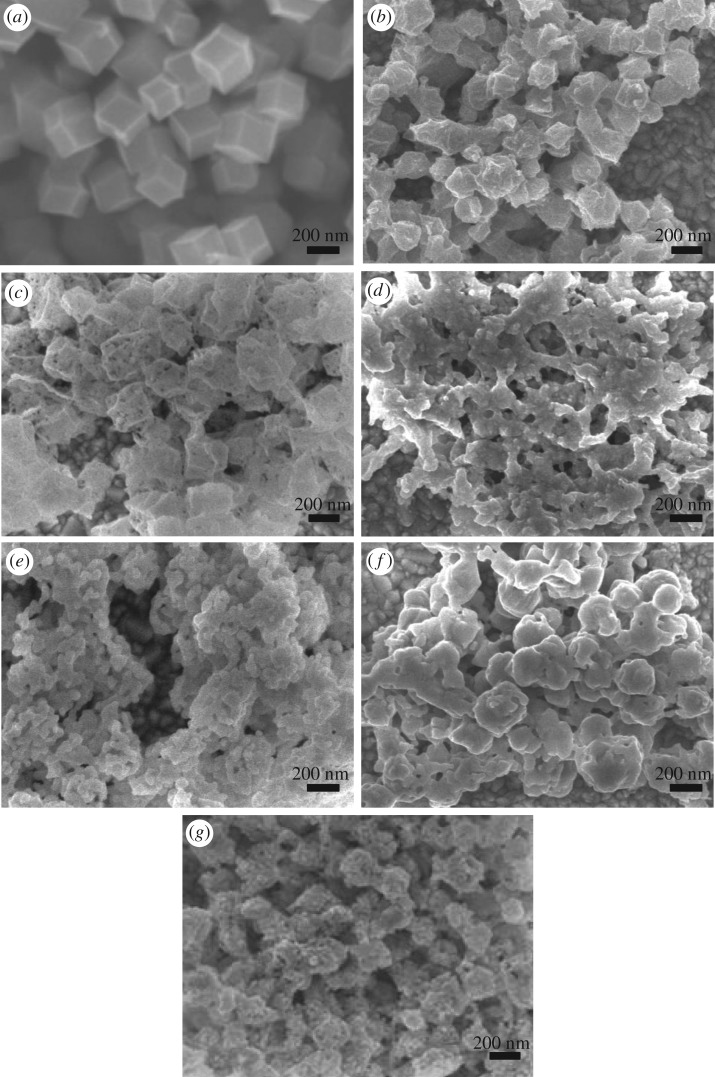
SEM surface images of the ZIF-67 thin film (*a*) and the ZIF-67 thin films carbonized for 15 min (*b*) and 30 min (*c*), sulfurated for 15 min (*d*), 30 min (*e*) and 60 min (*f*) and carbonized for 15 min followed by sulfuration of 15 min (*g*).

The morphologies of the calcinated ZIF-67 thin films were obviously decided by the calcinating time. It can be observed from SEM surface images of the thin films calcinated for different times, as shown in figure 1 and electronic supplementary material, figure S1. ZIF-67 has a rhombic dodecahedron metal framework structure, as shown in figure 1*a*. When the film was calcinated in N_2_ at 450°C, parts of H and O elements in the MOFs will be removed along with the N_2_ stream. The rhombic dodecahedron structure can be mainly kept, but the particle size became small for the 15 min carbonization (figure 1*b*), because after calcinated at 450°C for 15 min, the carbon skeleton shrinks sharply with the loss of other elements in the skeleton. Some black dots appear in the particles for the 30 min carbonization (figure 1*c*), and the colour of the nanocrystalline film turns from lavender to brown. The sulfuration treatment is the case where the sulfur vapour from sublimation S combines with ZIF-67 at 450°C. When the ZIF-67 thin film was calcinated under sulfur stream at 450°C for 15 min (figure 1*d*), the framework structure was partially destroyed to form some connected particles. The S adhered to the surface of the MOFs reacted with Co ions to produce CoS, and it also may react with carbon, oxygen and hydrogen to accelerate the collapse of framework. The porous structure of ZIF-67 was further destroyed to form some small particles after being sulfurated for 30 min (figure 1*e*). As the calcinating time prolongs to 60 min, the sulfur stream has more time to erode the MOF structure. The structure of the rhombic dodecahedron was almost collapsed as shown in figure 1*f*. As the edges and corners of the carbon skeleton disappeared, the particles became smaller and they became round. The particle diameter is approximately 300 nm. After the sulfuration for 120 and 180 min, the carbon skeleton was almost destroyed, and versatile porosities disappeared, MOFs were decomposed and became spherical or flaky shape (electronic supplementary material, figure S1). Moreover, the ZIF-67 thin film was carbonized for 15 min followed by the sulfuration for another 15 min (C15 + S15), and the surface morphologies show the porous rhombic dodecahedron (figure 1*g*). It roughly maintains the original size and structure, but some points on each particle were removed. As long as the time was further increased regardless of the carbonization or the sulfuration, the rhombic dodecahedron cannot be observed, and was replaced by small particles, as shown in electronic supplementary material, figure S1 (C15 + S30 and C30 + S30). In other words, there was enough time to destroy the carbon skeleton structure. Therefore, to reserve the porous framework of ZIF-67, the calcinating time should be less than 30 min.

**Figure 2.**
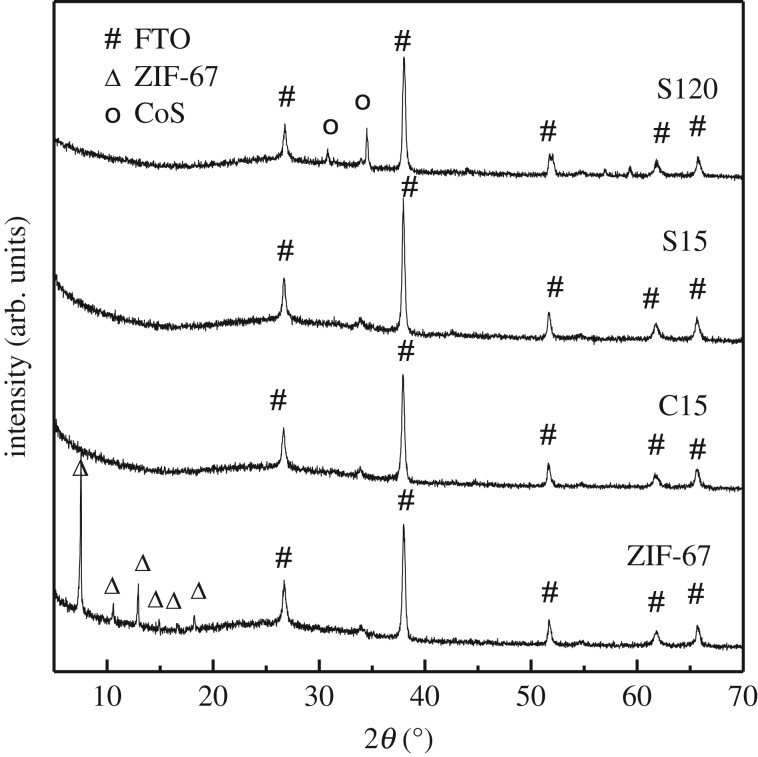
X-ray diffraction patterns of the ZIF-67 thin film and the ZIF-67-derived thin films prepared under different conditions.

X-ray diffraction patterns of ZIF-67 thin films calcinated at different conditions were measured to analyse the composition of the obtained materials [38]. Figure 2 and electronic supplementary material, figure S2 showed these XRD patterns. The XRD patterns of ZIF-67 coated on FTO glass show peaks at 7.57°, 10.57°, 12.96°, 14.90°, 16.69° and 18.26°, respectively, which are corresponding to the diffraction angles of (011), (002), (112), (022), (013) and (222) of ZIF-67 crystal plane according to the literature [21]. However, these corresponding XRD peaks disappeared after the carbonization for 15 min or sulfuration for 15 min, which proves that the crystalline structure of ZIF-67 was destroyed. The XRD peaks of samples from S15 to S180 (electronic supplementary material, figure S2a) are not basically changed, except that two peaks at 30.85° and 34.64° become more and more clear, which belong to the crystalline planes of (100) and (010) of CoS compared with the standard card of CoS. During the sulfuration treatment, CoS was generated accompanying the framework collapse of ZIF-67. The diffraction peaks of CoS become clear for S60 sample (electronic supplementary material, figure S2a), and they cannot appear as the calcinating time is less than 60 min (electronic supplementary material, figure S2b,c).

### 3.2. Catalytic activity of ZIF-67-derived thin films

**Figure 3.**
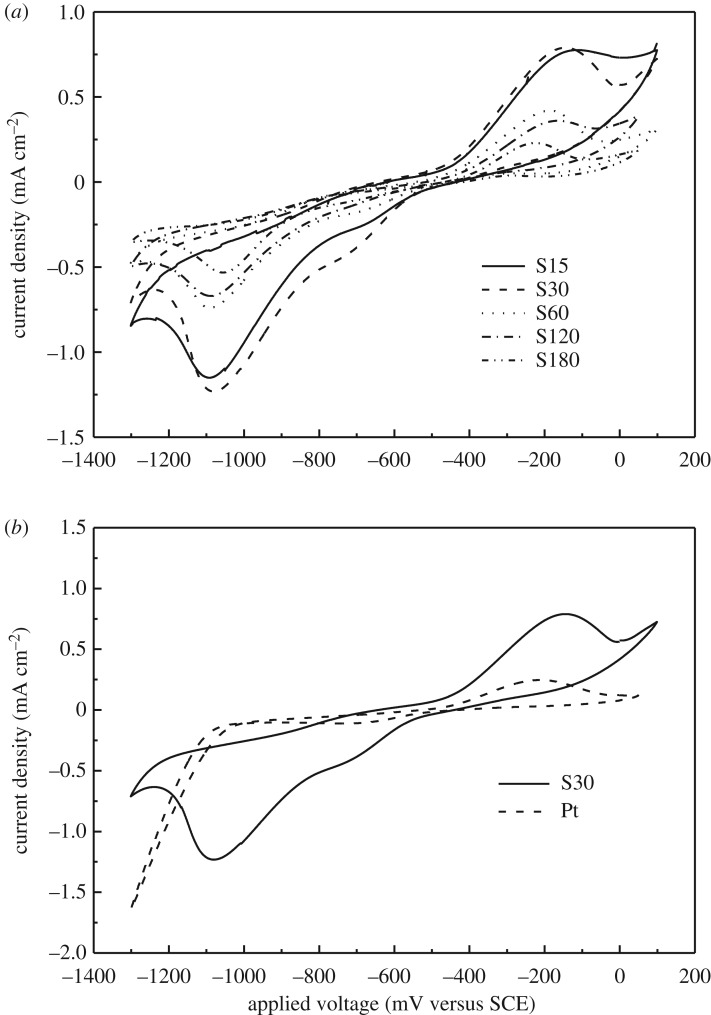
Cyclic voltammograms of the electrodes prepared by vulcanizing ZIF-67 thin film for different times (*a*) and Pt electrode and the electrode prepared by vulcanizing ZIF-67 thin film for 30 min (*b*). An aqueous electrolyte is 0.5 mM S, 2.0 mM Na_2_S and 0.2 mM KCl.

CV was measured to examine the electrocatalytic activity towards polysulfide redox electrolyte of the ZIF-67-derived CEs and Pt CE. The redox reaction processes of S^2−^/${{\text{S}}_{n}}^{2-}$ in polysulfide electrolyte can be described by analysing CV curves. Both oxidation and reduction peaks can be observed on the CV curves of the ZIF-67-derived electrodes prepared by the sulfuration, as shown in figure 3. As the reduction reaction of S*_n_*^2−^ to S^2−^ takes place at CE of QDSSCs, we will focus on the reduction part on CV curves for different ZIF-67-derived electrodes. As shown in figure 3 and electronic supplementary material, figure S3, there is a weak reduction peak at around potential of −0.70 V and a strong reduction peak at approximately −1.10 V, which can be regarded as the reduction peaks of the electrolyte on the surface of the electrode [38]. Moreover, the significant peaks cannot be observed in the vicinity of −0.70 V for samples of C15 and C15 + S15 (electronic supplementary material, figure S3). S30 sample shows the strongest reduction peaks, which means that the ZIF-67 thin film sulfurated for 30 min possesses the best electrocatalytic activity towards polysulfide solution. With further increasing the sulfuration time, the reduction ability generally exhibits a downward tendency according to the value and the position of the reduction peaks, as shown in figure 3. To evaluate the catalytic activity of S30 sample, CV curve of Pt electrode was measured and shown in figure 3*b*. At −0.70 V, there is a clear reduction peak, but it is smaller than that of the S30 sample. At more negative potential, there is no peak for Pt electrode; however, there is a bigger peak at −1.10 V for the S30 electrode. The ZIF-67 thin film sulfurated for 30 min exhibits better catalytic activity towards the reduction of polysulfide electrolyte than Pt.

**Figure 4.**
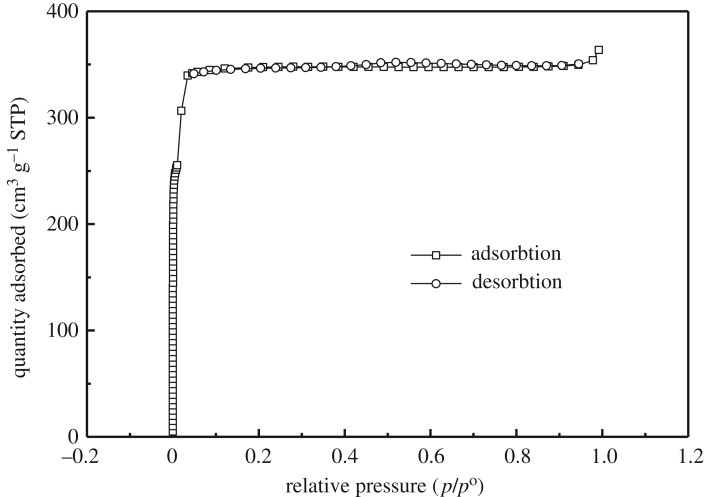
Nitrogen BET sorption isotherm of S30.

Low-temperature adsorption–desorption data indicate the formation of a developed microporous system and a high BET-specific surface area of the S30 sample (*S*_BET_ = 1018 m^2 ^g^−1^, *S*_Langmuir_ =1520 m^2^ g^−1^), as shown in figure 4. High-surface area can offer more active sites for the reduction process in polysulfide electrolyte. Therefore, CE with high-surface area will be beneficial to the efficiency improvement of solar cell. The big specific surface area is essential for the CE of QDSSCs.

### 3.3. Photovoltaic performance of QDSSCs based on ZIF-67-derived CEs

**Figure 5.**
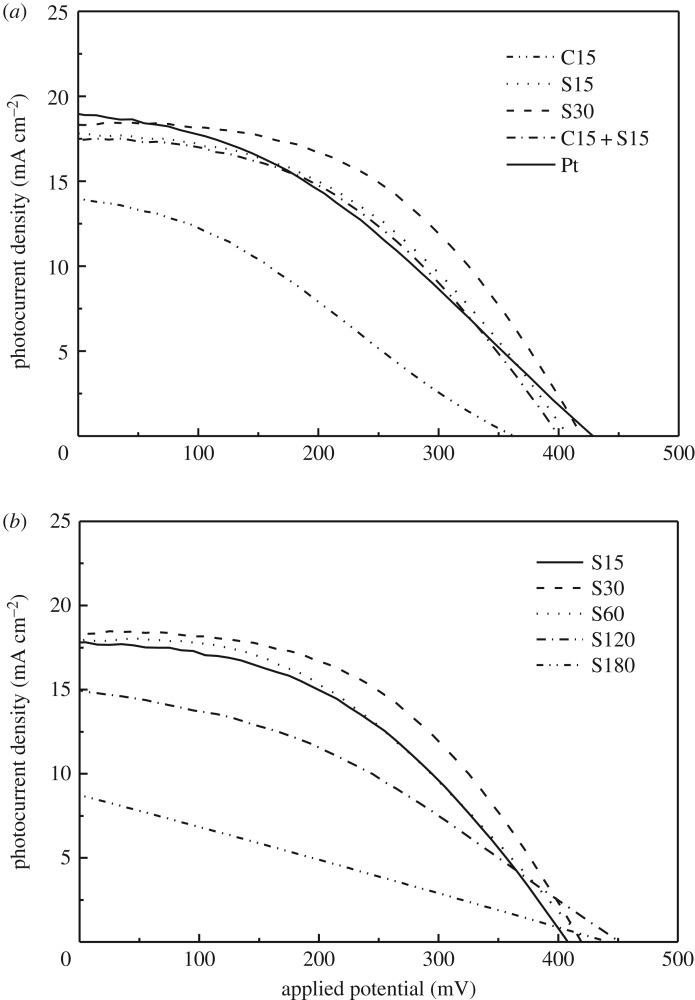
*J*–*V* curves of CdSe QDSSCs based on Pt and the ZIF-67-derived CEs prepared by different carbonization and sulfuration conditions (*a*) and by sulfuration for different times (*b*).

The ZIF-67-derived thin films on FTO by series of carbonization and sulfuration treatments were used as CEs to fabricate QDSSCs with CdSe QD-sensitized TiO_2_ nanocrystalline thin film as photoelectrode and polysulfide solution as electrolyte. Photocurrent–voltage curves for QDSSCs are shown in figure 5, and the corresponding photovoltaic parameters are given in table 1. With the higher specific surface area and catalytic activity, the S30 CE showed the highest light-to-electric conversion efficiency of 3.77% with *V*_oc_ of 0.42 V and *J*_sc_ of 18.5 mA cm^−2^ as expected. At the same time, the conversion efficiency of QDSSCs based on S15, S60, C15 + S15 and C15 + S30 is higher than that of QDSSC based on the Pt CE (2.98%), as shown in table 1. The carbonization sample of C15 shows worse performance than the sulfuration sample of S15 (figure 5*a*). The sulfuration is necessary to form the CE with high catalytic activity. With the increase of sulfuration time, the conversion efficiency of QDSSCs was first increased and then decreased after the maximum value of S30 CE (figure 5*b*). The extension of sulfuration time will destroy the porous structure as observed in SEM images. Therefore, there is the best sulfuration time for high catalytic activity. In this case, the sulfuration time is optimized to be 30 min.

**Table 1.** Photovoltaic parameters of QDSSCs based on ZIF-67-derived CEs and Pt.

CEs	*J*_sc_ (mA cm^−2^)	*V*_oc_ (mV)	*ff*	*η* (%)
C15	13.90	360	0.32	1.61
C30	11.30	370	0.21	0.88
S15	17.80	410	0.44	3.19
S30	18.50	420	0.48	3.77
S60	18.10	420	0.42	3.21
S120	15.10	450	0.39	2.89
S180	8.84	440	0.25	0.99
C15 + S15	17.50	400	0.44	3.10
C15 + S30	16.50	420	0.44	3.01
C30 + S30	16.80	400	0.41	2.75
Pt	18.90	430	0.37	2.98

### 3.4. Conversion enhancement mechanism of QDSSCs due to ZIF-67-derived CE

**Figure 6.**
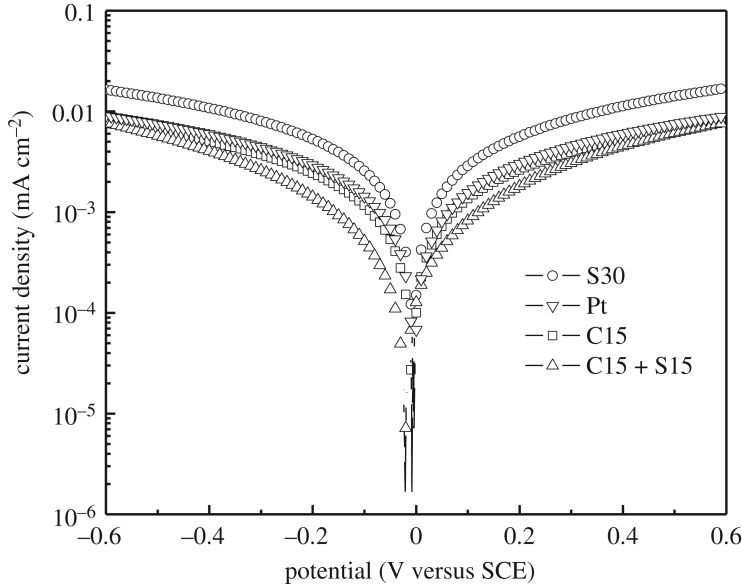
Tafel (semilog *V*–*C*) curves of Pt and the ZIF-67-derived CEs prepared by different carbonization and sulfuration conditions.

Voltage–current semilog (Tafel) curves of processed MOF thin films and Pt were measured to further indicate the enhancement mechanism of the conversion efficiency of QDSSCs due to the ZIF-67-derived CE and are shown in figure 6 and electronic supplementary material, figure S4. At the potential of 0.13 V, the corresponding current density values of S30, Pt, C15 and C15 + S15 are 3.7 × 10^−3^, 2.0 × 10^−3^, 1.7 × 10^−3^ and 1.1 × 10^−3^ mA cm^−2^, respectively. The conductivity of S30 is highest, which is one of the reasons for the highest conversion efficiency of QDSSC based on the S30 CE. For the sulfuration samples, the order of conductivity is S30 > S15 > S60 > S120 > S180 (electronic supplementary material, figure S4). As the sulfuration time is short, there are some organic ligands remaining in the porous thin film, which will hinder the movement of electrons leading to the low conductivity. However, as the sulfuration time is prolonged, the porous structure will be destroyed. Therefore, the highest conductivity of the ZIF-67-derived thin film can be obtained by sulfuration of 30 min. The high conductivity of CE is the premise for high conversion efficiency of QDSSCs.

**Figure 7.**
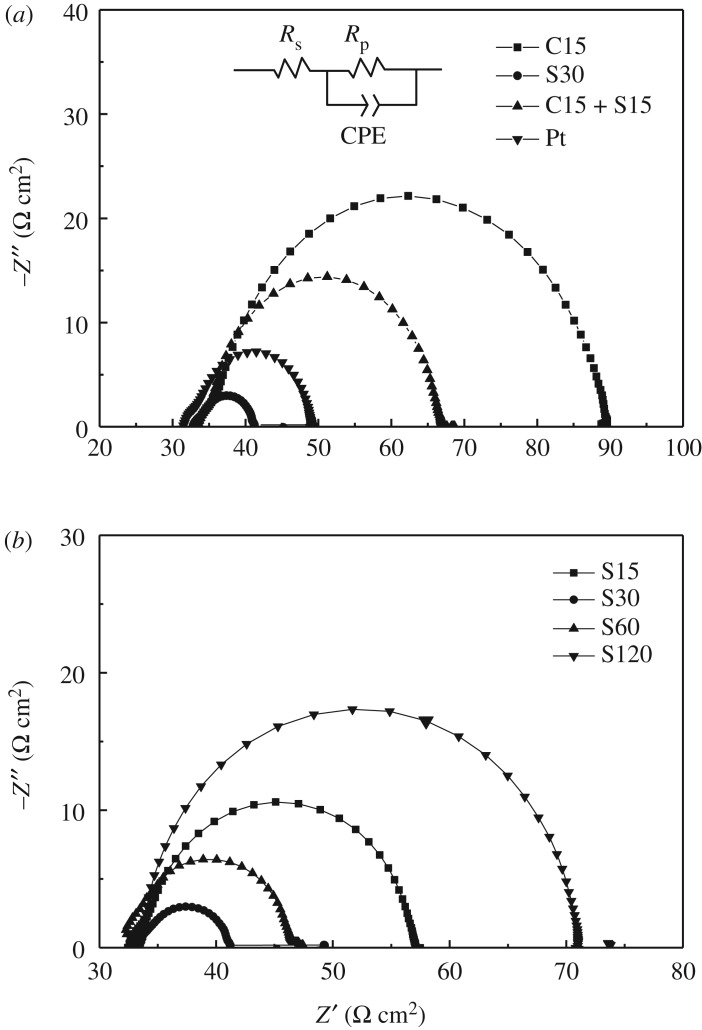
EIS of Pt and the ZIF-67-derived CEs prepared by different carbonization and sulfuration conditions (*a*) and by sulfuration for different times (*b*). The equivalent circuit to fit EIS is inset.

The interfacial charge transfer process is another factor to affect the conversion efficiency of QDSSCs. Through the measurement of EIS of CEs, the interfacial charge transfer resistance can be calculated. EIS of symmetrical cells based on two identical CEs was measured and is shown in figure 7. In the impedance, the series resistance (*R*_s_) is the intercept in real axis located in the high-frequency region, and the electrode/electrolyte interface charge transfer resistance (*R*_ct_) is located in the medium-frequency region. The electrocatalytic activity of the electrode towards polysulfide reduction can be evaluated according to the *R*_ct_ value, which is determined by fitting the Nyquist curve with the equivalent circuit [39]. The equivalent circuit to fit the curves is inset in figure 7, and the fitting data to the EIS were listed in table 2. *R*_s_ values are almost the same for all of samples because the resistance values of their substrates are almost the same. However, *R*_ct_ values are different; it is 11.97 Ω cm^2^ for S30, which is lower than that of Pt CE (17.10 Ω cm^2^). As the sulfurated time is increased, the variation trend of *R*_ct_ for ZIF-67-derived electrodes (as shown in figure 7*b*) is consistent with the result of Tafel measurement. The lowest *R*_ct_ value of S30 sample means that the interfacial charge transfer process is easy and thus leading to the best photovoltaic performance of QDSSC.

**Table 2.** Data fitted to EIS of QDSSCs based on ZIF-67-derived CEs and Pt.

CEs	*R*_s_ (Ω cm^2^)	*R*_ct_ (Ω cm^2^)	*C* (F cm^−2^)
C15	33.24	54.27	1.33 × 10^−4^
S30	32.81	11.98	3.70 × 10^−4^
C15 + S15	33.35	23.26	7.34 × 10^−5^
Pt	31.48	17.10	1.42 × 10^−4^
S15	32.44	21.73	2.49 × 10^−4^
S60	33.15	14.29	3.31 × 10^−4^
S120	32.60	31.70	6.19 × 10^−5^

## 4. Conclusion

The efficient ZIF-67-derived CE was prepared by simply vulcanizing ZIF-67 thin film. With the increase of sulfuration time, the porous framework of ZIF-67 thin films was gradually destroyed. Sulfuration of 30 min can convert the ZIF thin film into hybrid materials of carbon and CoS, and at the same time, the porous structure is mainly kept. The further measurements on the conductivity and interfacial charge transfer process for the sulfurated ZIF-67 thin film indicate that the ZIF-67-derived thin film by vulcanizing for 30 min has a low charge transfer resistance, a high BET-specific surface area and catalytic activity. QDSSC based on the ZIF-67-derived CE sulfurated for 30 min showed the best photovoltaic conversion efficiency of 3.77%, which is higher than that of the precious metal Pt CE one (2.98%). These results give a new idea of thinking about the future development of QDSSC.

## Supplementary Material

Supplementary material

## Data accessibility

The datasets supporting this article have been uploaded as part of the electronic supplementary material.
