# Supplementary material for: Zeolitic-imidazolate frameworks derived Pt-free counter electrodes for high-performance quantum dot-sensitized solar cells
Source: R Soc Open Sci. 2018 May 30;5(5):180335. doi: 10.1098/rsos.180335 (PMC5990812; doi:10.1098/rsos.180335)
Supplement: Supplementary material [file rsos180335supp1.doc]

**Zeolitic-imidazolate frameworks derived Pt-free counter electrodes for high performance quantum dot-sensitized solar cells**

**Supplementary Materials**


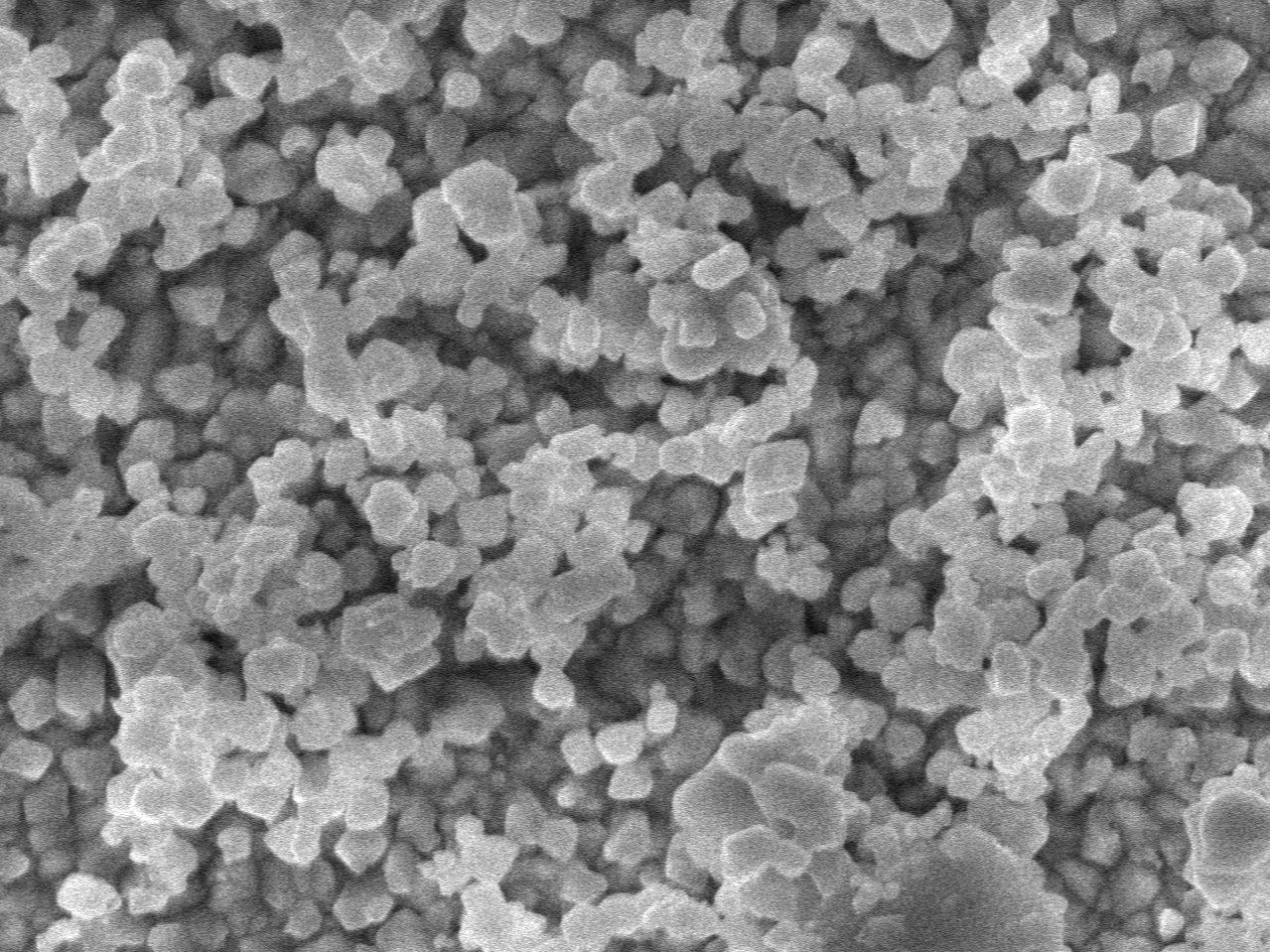

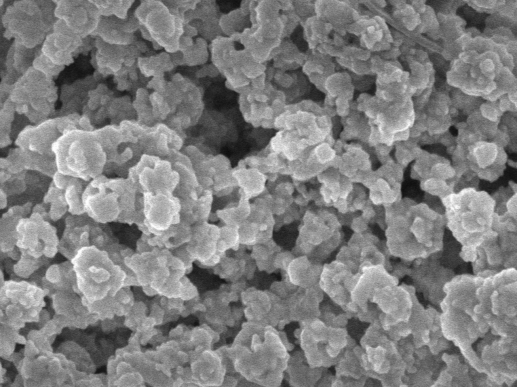

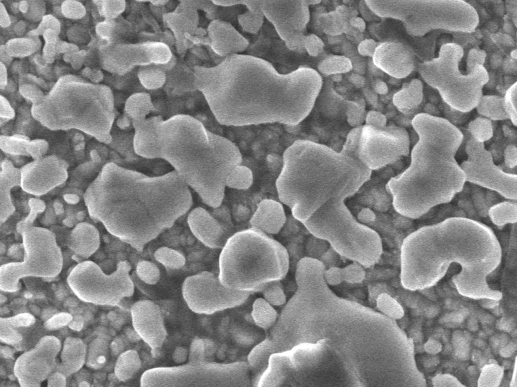

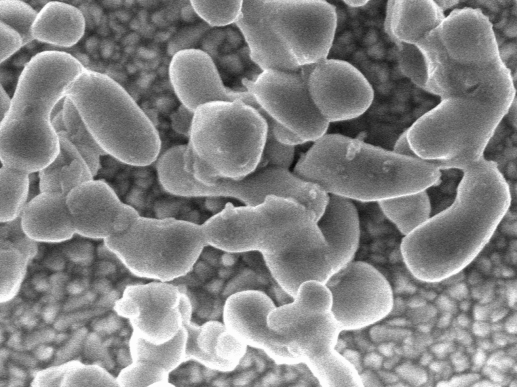


**100nm**

**a**

**100nm**

**c**

**100nm**

**b**

**d**

**100nm**

**Fig.S1** SEM surface images of the ZIF-67 derived thin films: (**a**) S120, (**b**) S180, (**c**) C15+S30 and (**d**) C30+S30.

**Fig.S2** XRD patterns of ZIF-67 and its derivative thin films.

**Fig.S3** Cyclic voltammograms of the ZIF-67 derived electrodes measured in an aqueous solution of 0.5 mM S, 2.0 mM Na_2_S and 0.2 mM KCl.

**Fig.S4** Tafel curves of the ZIF-67 derived electrodes.
